# Satisfaction With Governmental Risk Communication Both Increases and Decreases COVID-19 Mitigation Behaviours

**DOI:** 10.3389/ijph.2023.1604966

**Published:** 2023-03-01

**Authors:** Darrick Evensen, George Warren, Frederic Bouder

**Affiliations:** ^1^ Department of Politics and International Relations, University of Edinburgh, Edinburgh, United Kingdom; ^2^ King’s College London, London, United Kingdom; ^3^ Risk Management and Societal Safety Research Group, University of Stavanger, Stavanger, Norway

**Keywords:** risk communication, risk perception, COVID-19 testing, social distance, Europe, structural equation modelling

## Abstract

**Objectives:** Over 3 years of the COVID-19 pandemic, and intense societal and governmental response, a wealth of research has examined risk perceptions and public risk mitigation behaviours. The vast majority of this inquiry has focused on health risks. Nevertheless, as a “total social fact” influencing nearly every aspect of quotidian life, the pandemic engenders a wide range of risk perceptions.

**Methods:** *Via* a survey (N = 4,206) of representative samples of the general public in five European countries (Germany, Norway, Sweden, Switzerland, United Kingdom), we explore perceptions of a range of personal/public health, economic, and societal risks. We also investigate the effects of perceptions of official governmental risk communication in one’s country on risk perceptions and risk mitigation behaviours.

**Results:** Structural equation modelling reveals that whilst perceptions of effective risk communication directly increase behaviours that mitigate COVID-19 health risks, these same perceptions indirectly decrease behaviour frequency *via* a mediated relationship with societal risk perceptions.

**Conclusion:** The findings highlight the import of governmental authorities analysing and communicating about the range of risk perceptions citizens might have about a “total social fact” such as COVID-19.

## Introduction

The COVID-19 pandemic led countries to adopt a wide range of direct and indirect interventions. There has been much discussion about the effectiveness and efficiency of government interventions (1, 2). The vast amount of data that come as a by-product of the pandemic and its management offer a unique opportunity to conduct international comparisons (3), including *via* large-scale surveying (4). Research has explored how specific government measures and policies affect people’s behaviour (5–7), and how direct experience of government measures shape perception, satisfaction, and behaviour, including from patients and the general public (8).

Factors such as perceived quality of government interventions and policy expectations have been shown to influence satisfaction with policies (9). Conversely, pre-established views and levels of trust in government have also played a role in predicting expectations, acceptance, and compliance (4). Most of this comparative effort, however, builds from the premise that health risks are the main driver of perception and behaviour. This reflects a narrow interpretation of people’s concerns, as it tends to discard other apprehensions, such as economic and societal impacts linked to the pandemic and its management.

Early insights into people’s concerns suggest that other factors also shape risk mitigation behaviours, for instance life satisfaction (10). This lack of nuance is problematic because, beyond interventions focused on saving lives—from lockdowns to vaccination mandates, many support measures were also designed to reduce economic and social risks (e.g., furlough schemes). In this article, we present survey results that examine risk perceptions and communication preferences with a view to capture both personal/public health and under-researched (i.e., economic and societal) risks.

## Research Context

We examine risk perceptions and perceptions of governmental risk communication as predictors of COVID-19 risk mitigation behaviours. Properly tailored risk communication has long been seen as essential for encouraging uptake of behavioural responses to health concerns (11), and for shaping perceptions of risks (12, 13), with those perceptions also influencing health behaviours (14, 15). Good risk communication has been advanced as particularly necessary in relation to COVID-19 to respond to and provide material to counteract misinformation (16, 17). Furthermore, risk communication has been shown to affect COVID-19 risk mitigation behaviours directly, and as mediated through risk perceptions (18).

Risk perceptions have long been recognised as a central influence on self-protective behaviour (19). A meta-analysis of early literature reveals that perceived likelihood of being exposed to a health risk, perceived susceptibility to the health risk, perceived severity of the outcome, all combine to exert substantial influence on health behaviours (20). These studies focus heavily on a given individual’s perceptions of risks for their own health (21).

### Personal Health Risk Perceptions

Past studies frequently use generic definitions of risk perception, often relying on a single measure (22), as opposed to enumerating and operationalising the range of constructs that could be risk objects. This approach may overlook potential complexities and variance present when assessing a broader, more holistic perspective on pandemic risk perception. In the context of COVID-19, research has also tended to focus on the significant relationship between personal health risks and mitigation behaviours. For example, (23) find a significant relationship between perception of catching or dying from COVID-19 and increased likelihood of undertaking protective behaviours.

Similar findings of a relationship between perceptions of risk from COVID-19 and increased adherence with protective behaviours have been found across a range of studies (22, 24–27). Additionally, perceptions of mitigative measures themselves have been shown to influence behaviours (e.g., negative experience with masks) (28). Kittel et al. (29) highlight, with panel data from Austria, that social norms can increase mitigation behaviours even when personal health risk perceptions are low.

Despite the value of examining reactions to personal health risks (30, 31), this potentially neglects a more holistic understanding of COVID-19. Few, if any, objects in the modern world are as deserving of the label of “total social fact” as the coronavirus pandemic. What becomes “total” here are the ways in which people use the virus in communication and policy—the virus’s conceptualisation in social terms as a “pandemic,” including the various techniques and technologies used to mitigate its socially-labelled “risks.”

Mauss (32, 33) derived his idea of the “total social fact” from his work with his uncle Émile Durkheim. A total social fact as Mauss perceived it is based on Durkheim’s idea of a social fact—things that are: a) external to individuals (originated in collective phenomena rather than individual idiosyncrasies or psychology), b) systematic (going beyond situational variations) and c) sanctioning (it has a power and domain over individuals, if not coercive at least moral). A total social fact is all these things concerning certain phenomena that effectively cut through various domains of everyday life.

Mauss (32, 33) argues that a “total” social fact by definition changes everything about the previous collective setup in which the “fact” manifests. For example, a total social fact has an aesthetic impact (e.g., the visuality of signage and the imperative to wear masks), and definitely a legal impact too in the sense that it reorganizes moral dictums and also aids in spreading moral principles through society (e.g., debates on anti-vaxxers). Mauss further claims that if something is total it also has a spiritual impact on people’s social contracts (e.g., apocalyptic/millenarian trends on the one hand, or on religious authority used to combat “risky” behaviour on the other hand). Therefore, whilst no survey could fully capture all of the aspects of life entangled in a “total social fact,” we sought to at least expand to additional domains of risk beyond health.

“Total social fact” is particularly apropos considering Durkheim’s characterisation of totality as encompassing the three core areas of the social, psychological, and physiological (34). Durkheim (35) identified societal constructs that achieve this level of totality as perhaps the most important, yet understudied aspect of social systems. Vandenberghe and Véran (36) explore further the way in which COVID-19 can be characterised as a total social fact. Our research explores some of the less studied risk perceptions associated with this total social fact.

### Public Health Risk Perceptions

The COVID-19 pandemic has strongly affected public health, whether through comparatively greater mortality and/or infection rates, or knock-on effects on health services (37, 38). People understandably have concerns over their own health, but they may also have concerns about the health of people in their society more generally, and these risks could cause them to behave differently (39, 40). Pooling risk perception into variables focused on personal and public health risks, Dryhurst et al. (22) find high levels of risk perception and significant correlations with increased likelihood of undertaking protective behaviours. Similarly, Jørgensen et al. (41) find that individuals with increased perceptions of the threat of COVID-19 to themselves and close family or friends exhibited greater compliance with protective behaviours—perhaps related to the sanctioning capacity of a total social fact. Wise et al. (42) find that increased risk perception of COVID-19 infection was linked to greater likelihood of protective behaviours, but respondents assessed they were less likely than the average person to be infected with COVID-19.

### Personal Economic Risk Perceptions

Beyond health impacts, government responses and restrictions to manage COVID-19 transmission, hospitalisations, and deaths have fostered positive and negative economic impacts including job losses, furloughs, weak labour market, and worsening financial situations (43). As such, it may be relevant to evaluate the effect of individuals’ perceptions of the impact of COVID-19 on their own personal economic situation, and its relationship to protective behaviours. Indeed, the common narrative around the risks of COVID-19 has been predominantly focused on trade-offs between the health risks of the disease, and potential economic impacts of mitigating actions (44, 45). The systematic nature of the total social fact allows it carry over into domains beyond personal or even public health.

Siegrist et al. (46) and Ridenhour et al. (47) find that heightened economic risk perception is associated with greater likelihood of engaging with protective behaviours. Across multiple countries, Nisa et al. (48) find that heightened economic risk perception is a consistent predictor of increased protective behaviours and policy support. Conversely, Albrecht et al. (49) find that individuals who perceived COVID-19 as an economic risk were less likely to accept digital contact tracing applications. Despite these findings, little research has examined perceptions of economic risk to one personally, although Mahdavian et al. (50) do find that perceptions of personal economic risk in Germany (but not in the UK) negatively affect adoption of COVID-19 protective behaviours.

### Societal Risk Perceptions

The COVID-19 pandemic has also produced societal changes—arising from the external, systematic, and sanctioning nature of the total social fact. Risk perceptions of the societal impact of COVID-19 have received far less scholarly attention than health risk perceptions. These risks include concerns about national economic performance (debt, strength of economy/growth, unemployment rates, effects on small businesses (51), mental health and diminished education effects of children missing school (52–54), and restricted community and social connectivity (55–57). Another such risk involves concern about loss of trust in official authorities. When examining flood risk, Wachinger et al. (58) find a relationship between decreased trust in authorities and reduced willingness to undertake protective behaviours. In the context of COVID-19, German-speaking Swiss respondents with greater social trust in authorities displayed increased risk perceptions and were more likely to undertake protective behaviours (46). Nevertheless, in an eight-nation study, trust in institutions had little impact on protective behaviours during the first wave of the pandemic (41).

In a German study, respondents displayed greater public concern towards the idea of a recession resulting from COVID-19 than towards personal economic impacts (59). Early in the pandemic, Lanciano et al. (60) found that Italian respondents foresaw a greater probability of economic crisis due to COVID-19 than likelihood of contracting or dying from COVID-19. Despite these findings, we have not identified many studies focusing on non-economic and non-health risk perceptions.

### Risk Mitigation Behaviours

The primary reason risk perceptions matter from a public health perspective is their relationship to behaviours that could affect public health outcomes (59). For example, research has shown that perceived effectiveness of lockdowns is linked to greater non-adherence with recommended mitigation actions (61), and that perception of moral arguments and social norms supporting mitigation actions (62, 63) and perceived appropriateness of the rules (64) supports behaviour uptake. Indeed, the perceived need for mitigation behaviours, and their societal recommendation or requirement, is the sanctioning component of a total social fact.

Finally, because of the essential role of government communication in shaping, and potentially mitigating or exacerbating, risk perceptions of COVID, we sought to further understand how reactions to government communication efforts might influence risk perceptions, and in turn risk mitigation behaviours (18, 65).

### Research Questions

1. How do risk perceptions of COVID-19 compare across personal health, public health, personal economic, and societal risks?
2. To what extent do perceptions of governmental communication about COVID-19 affect behaviours that health authorities have recommended for limiting spread of COVID-19?
3. To what extent are any such effects mediated by risk perceptions associated with COVID-19?
4. How do the answers to the foregoing questions vary across the five European nations in our study?

## Methods

### Data Collection

We designed a survey to investigate: 1) public perceptions of risk communication about COVID-19, 2) sources they relied on for information about COVID-19, 3) perceptions of health, economic, and societal risks due to COVID-19, and 4) actions the survey respondents took in response to COVID-19. We ran the survey (N = 4,206) through the online survey panel provider Qualtrics from 1 April—4 May 2021 in five European countries (Supplementary Table S1). The sample was broadly representative in each nation (see Supplementary Methods). The median completion time was 19 min 27 s. Any respondents taking less than two-thirds of the median completion time were excluded from analysis as a speeding check.

Although there certainly were differences between the five countries in how members of the public perceived risks and responded to government communication, herein we focus on the similarities. Nuanced differences can be gleaned from Tables 4, 5, but the commonalities between countries speak to the overarching patterns that are less dependent on context specificity. We highlight those prominent patterns.

Our analysis herein focuses on three primary groups of variables, beyond demographic covariates. First, our exogenous attitudinal items of interest all relate to public perceptions of official governmental risk communication about COVID-19: 1) perceptions of the extent to which information needs were met, 2) extent to which the communication was clear and understandable, and 3) consistency in government recommendations. Complete wording of all survey items mentioned in this article is available in the Supplementary Methods.

Our second primary group of variables measured risk perceptions of 15 outcomes due to COVID-19. Three of these related to personal health risks, three to public health risks, three to personal economic risks, and six to societal risks (economic, political, and social). Exploratory factor analyses and reliability scaling reveals that the 15 items pool well onto these four domains. We discuss specific items further in the results section.

Our final set of key variables are behavioural outcomes. Because we are interested in the extent to which perceptions of official governmental risk communication shape actions that could mitigate the spread of COVID-19, we asked about frequency of two behaviours that were widely recommended by public health experts throughout the pandemic in each of the five countries in our study: “keeping the required social distance” and “getting tested when having symptoms.”

### Data Analysis

**FIGURE 1:**
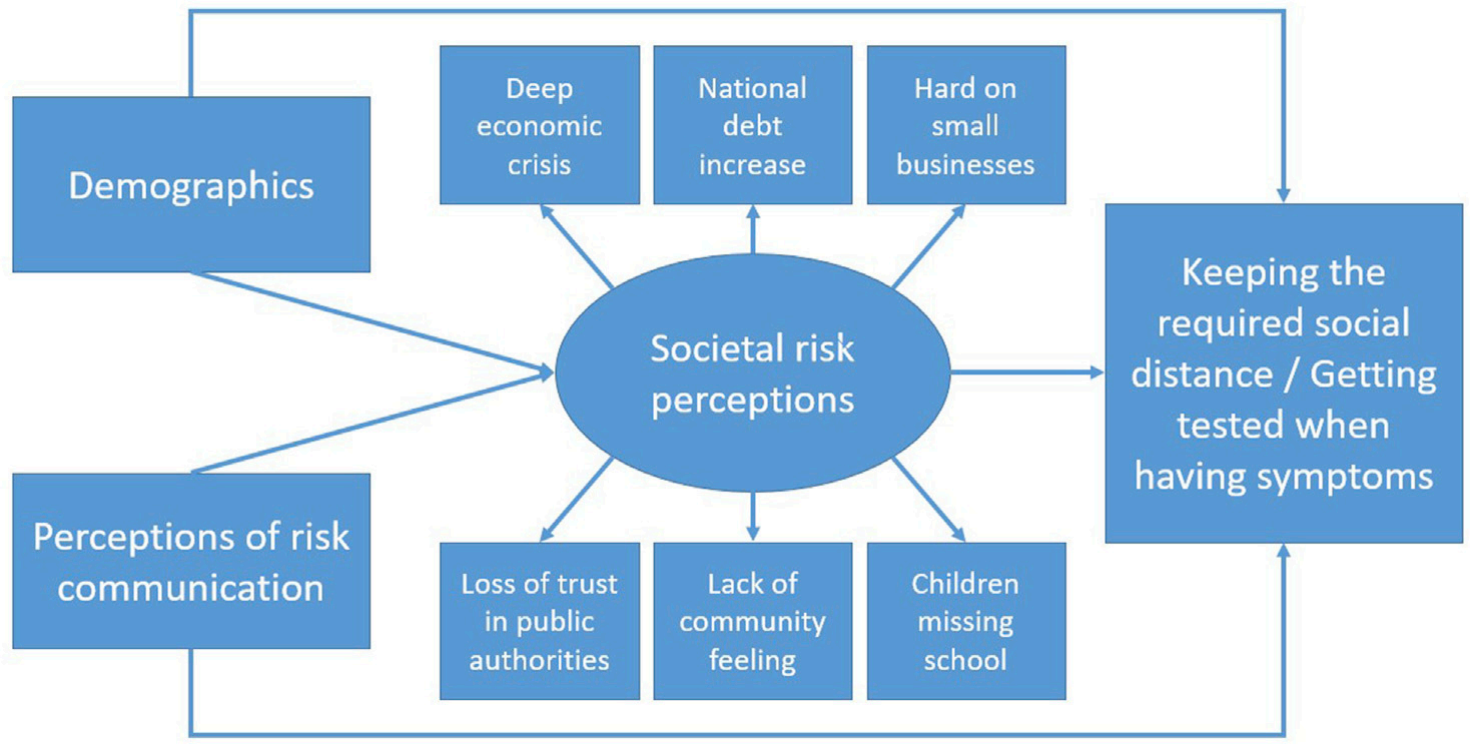
General form of the structural equation model.

We explore our first research question *via* basic descriptive statistics, repeated measures ANOVAs to compare across risk perceptions, and bivariate correlations examining relationships between risk perceptions and risk mitigation behaviours. We answer the next three research questions is *via* a series of structural equation models (SEMs) that combine confirmatory factor analyses with structural regression pathways. We chose this approach due to our theoretically constructed latent variable of societal risk perceptions (composed of six measured variables) and the multi-stage mediated regression pathways needed to answer our questions. We chose to focus on the role societal risk perceptions, because the literature review revealed that research to date on COVID-19 risk perceptions has given the least attention to these risks. Figure 1 offers a general depiction of the model.

We ran separate models for the respondents from each country in our study, and then one combined model for the full sample (N = 4,206). We ran this set of six models for each of the two dependent variables of interest: respondents’ frequency of “keeping the required social distance” and “getting tested when having symptoms”; therefore, twelve SEMs in total. The direct pathway in Figure 1 from “perceptions of risk communication” to the two COVID-19 mitigation behaviours addresses RQ2, the indirect pathway between these two variables (through societal risk perceptions) speaks to RQ3, and comparison of the five country-specific sub-sample SEMs for each dependent variable investigates RQ4.

In addition to the SEMs, we include some basic independent samples t-tests, bivariate correlations, chi-square tests on crosstabs, and ANOVAs to explore socio-demographic predictors of perceptions of risk communication and to assess the relationship between the multiple types of risk perceptions we measured in the survey.

## Results

### Risk Perceptions

We measured four broad content foci for risk perceptions: personal health risk, public health risks, personal economic risks, and societal risks (economic, political, social). Initial exploratory factor analyses revealed clear pooling of the fifteen measured items into the four aforementioned categories (see Supplementary Methods for all items and reliability coefficients).

To examine our first research question, we first ran repeated measures ANOVAs to compare the means of the personal health and personal economic risk items (Table 1), and then the public health and societal risk items (Table 2). Results reveal that on aggregate, percentage chance of contracting COVID-19 and of financial situation worsening were the most commonly perceived personal health and economic risks. The percentage chances in Table 1 varied moderately from one another (eta^2^ effect size = 0.11). Perceptions of public health and societal risks on average fell between moderate and significant levels of risk; the lowest risk estimate was for more people dying in one’s country compared to elsewhere, and the highest estimate for the pandemic being hard on small and medium size businesses. The risk perceptions in Table 2 varied moderately (eta^2^ = 0.15).

**TABLE 1:** Average percentage perceived chance of personal risk outcomes (Germany, Norway, Sweden, Switzerland, United Kingdom, April 2021).

	Arithmetic mean	Standard deviation
% chance you will get COVID-19*	26.6^a^	22.6
% chance you will be hospitalised because of COVID-19	16.8^b^	20.7
% chance you will die from COVID-19	12.0^c^	19.9
% chance your financial situation will worsen	25.2^d^	29.1
% chance you will lose your job	13.0^c^	23.2
% chance your relatives or family members will lose their jobs	19.7^e^	23.7

*All items measure the perceived percentage chance this will happen in the next 3 months.

^a^ Superscript letters that differ connote significant differences at *p* < 0.05 (based on a repeated measures ANOVA). Values with the same letter do not differ significantly.

**TABLE 2:** Average perception levels for public health and societal risks in your country (Germany, Norway, Sweden, Switzerland, United Kingdom, April 2021).

	Arithmetic mean	Standard deviation	“Don’t know” (%)
More people falling ill than elsewhere*	3.05^a^	1.1	2.5
More people dying than elsewhere	2.92^b^	1.1	2.7
Health services overstretched	3.69^c^	1.1	1.1
Deep economic crisis	3.39^d^	1.1	1.9
National debt increase	3.74^c^	1.1	2.9
Hard on small and medium size businesses	4.07^e^	0.9	1.3
Loss of trust in public authorities	3.56^f^	1.1	2.2
Lack of community feeling and solidarity	3.42^d,g^	1.1	2.5
Children missing school	3.47^g^	1.1	1.7

*All items were measured on a 1–5 scale, with a “do not know” option: 1 = no risk at all, 2 = low level of risk, 3 = moderate level of risk, 4 = significant risk, 5 = severe risk. To calculate means, we treat “do not know” responses as missing data.

^a^ Superscript letters that differ connote significant differences at *p* < 0.05 (based on a repeated measures ANOVA). Values with the same letter do not differ significantly.

### Risk Perceptions as Mediator

To answer our second research question (risk perceptions mediating between views of governmental risk communication and mitigation behaviours), we chose to include the risk perceptions that have been least discussed in academic literature on COVID-19. Initial exploratory correlations also revealed moderately strong negative correlations between perceptions of risk communication and societal risk perception—suggesting an intriguing relationship opposite to that of risk communication perceptions on health risk perceptions (Table 3).

**TABLE 3:** Bivariate correlations between selected risk perceptions, beliefs about risk communication, and COVID-19 mitigation behaviours (Germany, Norway, Sweden, Switzerland, United Kingdom, April 2021).

	Clear and understandable communication	Consistent communication	Keep required social distance	Test when have symptoms
Chance you get COVID-19	0.11*	0.13*	0.17*	0.20*
Chance you are hospitalised	0.12*	0.12*	0.13*	**0.11**
More people fall ill here	0.04	0.04	0.21*	0.26*
Health services overstretched	−0.02	−0.03	0.20*	0.26*
Financial situation worsen	−0.02	−0.01	0.02	−0.01
Chance of losing your job	−0.04	0.07*	−0.05	0.04
Hard on small businesses	−0.09*	−0.11*	0.10*	0.21*
Loss of trust in authorities	−0.19*	−0.21*	0.00	0.07

*Bold values denote statistically significant correlations at *p* < 0.05; an asterisk (*) denotes *p* < 0.01.

Our subsequent analyses all rely on the “societal” sub-set of risk perceptions. In Table 3, the public health risk perceptions are clearly particularly important for explaining COVID-19 mitigation actions; nevertheless, this would be expected intuitively—due to the ability of COVID-19 mitigation actions to improve public health outcomes—and empirically—based on the studies reviewed previously.

### Communication’s Effect on Risk Perceptions and COVID-19 Mitigation Behaviours

We ran twelve SEMs to assess our research questions. Table 4 presents the results of the six SEMs—one for each country and one for the full sample—with “keeping the required social distance” as our final outcome variable. Table 5 presents the results of the six SEMs with “getting tested when having symptoms” as our outcome variable.

**TABLE 4:** Structural equation model predicting “keeping the required “social distance,”” with social risk perceptions as a mediator (Germany, Norway, Sweden, Switzerland, United Kingdom, April 2021).

	Germany (risk R^2^ = 0.04 dist. R^2^ = 0.20)	Norway (risk R^2^ = 0.16 dist. R^2^ = 0.12)	Sweden (risk R^2^ = 0.15 dist. R^2^ = 0.11)	Switzerland (risk R^2^ = 0.18 dist. R^2^ = 0.16)	UK (risk R^2^ = 0.05 dist. R^2^ = 0.12)	Full sample (risk R^2^ = 0.13 dist. R^2^ = 0.10)
Effect on societal risk perceptions						
Demographics						
Age	—	—	−0.09*	—	−0.12**	—
Sex (female = 0, male = 1)	—	−0.09	−0.12**	—	—	−0.09**
Education (at least BA = 1, less = 0)	—	—	—	—	0.06	−**0.04**
Income	—	−**0.07**	—	—	—	n/a^a^
Political orientation (1 = very left wing, 7 = very right wing)	—	—	**0.08**	**0.09**	—	—
Born in country? (1 = Yes)	—	—	—	—	—	—
Belong to any religion/faith? (1 = Yes)	—	—	—	−0.15**	—	—
City size (higher = larger population)	—	**0.08**	—	—	—	**0.04**
Believe you had COVID (1 = Yes)	—	—	—	—	—	0.06*
Risk communication						
Authorities met need for information?	—	—	−0.06	−0.17**	—	−0.14**
Official messages clear, understandable	—	−0.17*	—	—	—	−0.08*
Official recommendation consistent?	—	−0.24**	−0.27**	−0.30**	—	−0.22**
Effect on keeping the required social distance						
Demographics						
Age	0.30**	—	0.14**	0.25**	0.18**	0.18**
Sex (female = 0, male = 1)	−0.15**	—	-0.19**	—	−0.11*	−0.10**
Education (at least BA = 1, less = 0)	**0.11**	—	—	0.08	—	—
Income	—	—	—	**0.10**	—	n/a^a^
Political orientation (1 = very left wing, 7 = very right wing)	—	—	—	0.08	−**0.09**	—
Born in country? (1 = Yes)	—	—	**0.11**	—	—	—
Belong to any religion/faith? (1 = Yes)	—	—	−**0.10**	—	—	−**0.05**
City size (higher = larger population)	—	—	—	−**0.10**	—	—
Believe you had COVID (1 = Yes)	—	—	—	—	—	−**0.05**
Risk communication						
Authorities met need for information?	**0.16**	0.19*	0.09	0.12	**0.11**	0.15**
Official messages clear, understandable	—	0.17*	−0.10	—	—	—
Official recommendation consistent?	**0.16**	—	—	**0.17**	0.23**	0.11**
Societal risk perceptions (latent var.)	—	0.07	—	**0.12**	—	0.07**
Model Fit Indices						
RMSEA	0.065	0.037	0.052	0.046	0.045	0.044
CFI	0.833	0.944	0.878	0.908	0.927	0.929
SRMR	0.052	0.029	0.034	0.033	0.033	0.024
Chi-square (d.f.)	171.3 (67)	126.0 (67)	187.3 (67)	132.2 (67)	153.0 (67)	391.1 (62)

Note: vacant cells denote statistical significance of *p* > 0.10; **bold** denotes statistical significance at least at *p* < 0.05, one * for *p* < 0.01, two ** for *p* < 0.001; values in the table represent standardised beta coefficients.

^a^ Income was measured on different scales in each country, due to different currencies and distributions of income in the population; therefore, this variable is excluded from the full sample analysis.

**TABLE 5:** Structural equation model predicting “getting tested when having symptoms,” with social risk perceptions as a mediator (Germany, Norway, Sweden, Switzerland, United Kingdom, April 2021).

	Germany (risk R^2^ = 0.04 test R^2^ = 0.16)	Norway (risk R^2^ = 0.16 test R^2^ = 0.11)	Sweden (risk R^2^ = 0.14 test R^2^ = 0.07)	Switzerland (risk R^2^ = 0.19 test R^2^ = 0.11)	UK (risk R^2^ = 0.07 test R^2^ = 0.12)	Full sample (risk R^2^ = 0.13 test R^2^ = 0.06)
Effect on societal risk perceptions						
Demographics						
Age	—	—	−0.09*	—	−0.14**	—
Sex (female = 0, male = 1)	—	−0.09	−0.12**	—	—	−0.09**
Education (at least BA = 1, less = 0)	—	—	—	—	0.08	−0.04
Income	—	−0.07	—	—	—	n/a^a^
Political orientation (1 = very left wing, 7 = very right wing)	—	—	0.08	0.09	—	—
Born in country? (1 = Yes)	—	0.06	—	—	—	—
Belong to any religion/faith? (1 = Yes)	—	—	—	−0.15**	0.05	—
City size (higher = larger population)	—	0.08	—	—	—	0.04
Believe you had COVID (1 = Yes)	—	—	—	—	—	0.06*
Risk communication						
Authorities met need for information?	—	—	−0.06	−0.18**	−0.13*	−0.14**
Official messages clear, understandable	—	−0.17**	—	—	—	−0.08*
Official recommendation consistent?	—	−0.23**	−0.27**	−0.30**	—	−0.22**
Effect on testing when displaying symptoms						
Demographics						
Age	0.13	—	−0.09	—	0.10	—
Sex (female = 0, male = 1)	−0.13	−0.10	−0.22**	—	−0.09	−0.08**
Education (at least BA = 1, less = 0)	—	—	—	—	—	0.05
Income	0.18*	—	—	—	0.23	n/a^a^
Political orientation (1 = very left wing, 7 = very right wing)	−0.13	—	—	—	−0.09	−0.05
Born in country? (1 = Yes)	—	0.16**	—	—	—	0.05
Belong to any religion/faith? (1 = Yes)	—	—	−0.07	—	—	—
City size (higher = larger population)	—	—	−0.09	—	—	—
Believe you had COVID (1 = Yes)	—	-0.11*	—	—	—	−0.04
Risk communication						
Authorities met need for information?	0.19**	0.14**	—	0.15*	0.15**	0.14**
Official messages clear, understandable	—	—	—	—	—	0.08
Official recommendation consistent?	—	—	—	0.19*	0.14	0.07
Societal risk perceptions (latent var.)	—	0.09	—	0.12	0.11	0.06
Model Fit Indices						
RMSEA	0.067	0.040	0.053	0.045	0.045	0.043
CFI	0.832	0.942	0.882	0.917	0.931	0.930
SRMR	0.052	0.030	0.033	0.032	0.035	0.024
Chi-square (d.f.)	178.6 (67)	138.0 (67)	190.8 (67)	128.7 (67)	155.6 (67)	378.1 (62)

Note: vacant cells denote statistical significance of p > 0.10; **bold** denotes statistical significance at least at *p* < 0.05, one * for *p* < 0.01, two ** for *p* < 0.001; values in the table represent standardised beta coefficients.

^a^ Income was measured on different scales in each country, due to different currencies and distributions of income in the population; therefore, this variable is excluded from the full sample analysis.

In the SEMs of the full survey sample (final column in Tables 4, 5), perceptions of communication from official authorities predict social distancing and testing behaviour. Beliefs that information needs have been met and that messages are consistent lead to higher frequency of undertaking the two COVID-19 mitigation behaviours (Tables 4, 5).

These positive relationships are unsurprising. However, these same risk communication beliefs *reduce* societal risk perceptions. Because societal risk perceptions increase COVID-19 mitigation actions (Tables 4, 5), this equates to a *mediated indirect effect* of official risk communication reducing COVID-19 mitigation behaviour. For example, increasingly positive perceptions of risk communication lead to lower societal risk perceptions, and then these lower societal risk perceptions lead to reduced frequency of undertaking the two COVID-19 mitigation behaviours we analysed.

The direct and indirect effects of beliefs about official authorities’ communication on COVID-19 mitigation behaviours mean that concurrently positive beliefs about communication are *directly increasing* and *indirectly decreasing* frequency of mitigation behaviours. The direct effect is admittedly larger than the indirect effect (0.11 vs. −0.02 for the effect of consistent messaging on social distance, and 0.07 vs. −0.01 for its effect on testing, in the full sample); nevertheless, the indirect is significant.

### Differences Across the Five Countries

There are clear differences cross-nationally in the goodness-of-fit of the SEMs, and in the coefficient values and statistical significance of key structural regression pathways in the models. Norway, Switzerland, and the UK have good overall model fit, whereas Germany and Sweden do not [see notably the CFI (66)]; this applies for both COVID-19 mitigation behaviours.

Although there are some variations in which pathways between variables are significant across the countries, the risk communication variables have fairly consistent effects in Norway and Switzerland on societal risk perceptions and on both of the COVID-19 mitigation behaviours. In Sweden, beliefs about risk communication are relevant for predicting societal risk perceptions, but not notably so for predicting COVID-19 mitigation action. The reverse is true in the UK and Germany—beliefs about risk communication are relevant for predicting COVID-19 mitigation actions, but not notably so for predicting societal risk perceptions.

Importance of demographics varies more widely between countries. The effects of socio-demographics on societal risk perceptions and risk mitigation behaviours are reflected in Tables 4, 5. We further examined the potential effects of a large range of socio-demographic variables on perceived consistency of risk communication and whether needs for information on COVID-19 were met or not (Supplementary Tables S2, S3). Across both perceptions of governmental risk communication, the clearest message is that few of the socio-demographic variables proved significant, and that there is little congruence across countries. Germany had the largest number of significant predictors of communication perceptions.

Being male was the most common predictor cross-nationally of viewing governments as providing no useful information. Political orientation was the most common predictor of perceived consistency of governmental communication, although the direction of effect varied—right-leaning associated with greater consistency in Norway and the UK (governed by Conservative parties during April 2021 survey administration) and left-leaning in Sweden and Switzerland (governed by the left-leaning Social Democrats in Sweden and Switzerland). Switzerland was governed by a coalition during survey administration, but Alain Berset, the Head of the Federal Department of Home Affairs—a leading spokesperson for the Swiss response to the pandemic, is a Social Democrat.

## Discussion

Our finding that most advances literature of risk perceptions and risk mitigation actions in relation to COVID-19 is the nuanced relationships in the mediated pathway from perceptions of risk communication to societal risk perceptions then to behaviours. Perceptions of good risk communication (i.e., governmental authorities being consistent, and meeting information needs) directly increases risk mitigation behaviours. In other words, perceptions of good quality risk communication leads to support for sanctioning in response to the total social fact of COVID. Indirectly, however, such perceptions lessen risk mitigation behaviours, mediated *via* decreased societal risk perceptions.

In this latter instance, put in the concepts of the total social fact, the reduced risk perceptions seem to, indirectly, reduce perceptions of the external threat to certain valued objects, and thus perhaps necessitate less sanctioning. Additionally, the heavy focus on health in most risk communication on COVID from governmental authorities could be seen as reducing the “totality” of the total social fact, because there is not strong focus on it cutting across multiple domains of everyday life. Furthermore, thinking government communication is good quality could foster social norms to support mitigative actions (29), but social norms and trust in information sources have also been shown to decrease certain risk perceptions about COVID (67).

The mediated indirect reduction in risk mitigation behaviours highlights the importance of providing attention in risk communication to risks other than solely health risks. One feasible interpretation of our data is that effective government communication about health risks crowded out other concerns. Societal risk perceptions, however, also associate positively with risk mitigation behaviours, suggesting that efforts to highlight those risks could be further beneficial for government entities seeking to promote risk mitigation actions. This is consistent with recent research revealing that social histories and myths concerning masking in societies have heavily influenced the political nature of relevant mitigation actions (68). Discourse about risk mitigation behaviours shapes risk perceptions, and then adoption of those behaviours.

Beyond seeking to influence specific behaviours, one might argue from a normative perspective that a key role of government is to provide its citizens with the resources to make informed decisions broadly, and that a more holistic treatment of risks from a “total social fact” such as COVID-19 would help facilitate such decision making (17). Social constructions of what the virus is and what it means for our lives (e.g., in health messaging and policy) have become a defining principle of risk communication and quotidian risk perception for virtually everyone in the societies we analyse herein. Governments, and other communicators, must remain cognisant that all actions such as testing when having symptoms and social distancing cut across domestic and public spaces, and across economic, social, cultural, religious, political, and indeed health dimensions. The totality of social constructions of the pandemic cannot be reduced to a single dimension in academic research or public communication. Perhaps communication should explicitly seek to afford attention to Durkheim’s components of the social fact—it being external, systematic, and sanctioning—and Mauss’s characterisation of totality—cutting across multiple domains of quotidian existence (36).

Our series of structural equation models reveal some overarching patterns, but also clear variation cross-nationally. Our core messages herein about relationships between governmental risk communication, societal risk perceptions, and risk mitigation behaviours apply most clearly in our Norway, Switzerland, and UK samples. For Germany and Sweden, additional empirical research and/or revised theoretical models are needed to better understand the weak relationships between these variables.

In summary, our five-nation survey reveals the import of governmental authorities remaining cognisant of and vigilant to the range of risk perceptions citizens might have about a “total social fact” such as COVID-19. The pandemic, the societal response, and the ways in which individuals reacted touched nearly every aspect of quotidian life. We have not systematically analysed governmental risk communication; neither are we seeking to critique any governmental authority for neglecting specific risks. We are, rather, cautioning that risk communication attending exclusively to individual and/or public health risks might foster unintended consequences for risk mitigation behaviours. A total social fact could clearly impact upon a range of risks and concerns, extending well beyond the six societal risks included in our models. Yet, these provide a start for conversations on how to expand the suite of COVID-19 relevant risks.
