# Supplementary material for: Satisfaction With Governmental Risk Communication Both Increases and Decreases COVID-19 Mitigation Behaviours
Source: Int J Public Health. 2023 Mar 1;68:1604966. doi: 10.3389/ijph.2023.1604966 (PMC10014469; doi:10.3389/ijph.2023.1604966)
Supplement: Supplementary file 1 [file Table1.docx]

Supplemental Table 1. Demographic distribution of the survey sample (percentages)

|  | **Norway**  N=855 | **Sweden**  N=845 | **Switzerland**  N=832 | **Germany**  N=835 | **United Kingdom**  N=839 |
| --- | --- | --- | --- | --- | --- |
| **Age:** | | | | | |
| 18-29 years | 21 | 19 | 17 | 16 | 20 |
| 30-39 years | 20 | 19 | 19 | 14 | 17 |
| 40-49 years | 19 | 15 | 17 | 18 | 19 |
| 50-59 years | 17 | 15 | 18 | 15 | 16 |
| 60-69 years | 14 | 16 | 15 | 24 | 14 |
| 70+ years | 9 | 16 | 14 | 13 | 14 |
| **Sex:** | | | | | |
| Female | 50.4 | 50.3 | 50.0 | 50.4 | 50.4 |
| Male | 49.5 | 49.2 | 49.8 | 49.4 | 48.5 |
| Trans / non-binary / other | 0.1 | 0.5 | 0.2 | 0.2 | 1.1 |
| **Education:** | | | | | |
| Less than secondary | 7 | 10 | 1 | 1 | 5 |
| Secondary education | 28 | 35 | 22 | 18 | 59 |
| Technical diploma / apprenticeship | 18 | 16 | 44 | 54 | 8 |
| Bachelors / undergraduate | 31 | 26 | 16 | 11 | 19 |
| Masters / postgraduate degree | 14 | 11 | 14 | 14 | 7 |
| PhD or equivalent | 2 | 2 | 3 | 2 | 2 |
| **Income:** | | | | | |
| 0-256,000 NOK; 0 SEK;  0-26,000 CHF; 0-10,799 €; £0-12,999 | 15 | 5 | 17 | 8 | 14 |
| 256,000-452,000 NOK; 1-149,000  SEK; 26,001-52,000 CHF; 10,800-  18,000 €; £13,000-18,999 | 25 | 15 | 22 | 15 | 12 |
| 452,001-666,000 NOK; 150,000-  299,999 SEK; 52,001-78,000 CHF;  18,001-24,000 €; £19,000-25,999 | 25 | 22 | 26 | 15 | 14 |
| 666,001-986,000 NOK; 300,000-  399,999 SEK; 78,001-104,000 CHF;  24,001-38,400 €; £26,000-31,999 | 20 | 23 | 23 | 28 | 12 |
| 986,001-1,526,000 NOK; 400,000-  499,999 SEK; 104,001+ CHF;  38,401-54,000 €; £32,000-47,999 | 12 | 17 | 12 | 17 | 21 |
| 1,526,000+ NOK; 500,000-699,000  SEK; 54,001-72,000 €; £48,000-  63,999 | 3 | 13 | -- | 10 | 13 |
| 700,000+ SEK; 72,001+ €;  £64,000-95,999 | -- | 5 | -- | 7 | 9 |
| £96,000+ | -- | -- | -- | -- | 5 |
| **Born in this country:** | | | | | |
| Yes | 88 | 89 | 81 | 93 | 92 |
| No | 12 | 11 | 19 | 7 | 8 |
| **City size**: |  |  |  |  |  |
| Fewer than 5,000 people | 16 | 12 | 27 | 17 | 16 |
| 5,000 – 9,999 | 11 | 7 | 20 | 9 | 8 |
| 10,000 – 49,999 | 24 | 22 | 25 | 25 | 21 |
| 50,000 – 99,999 | 15 | 14 | 6 | 13 | 11 |
| 100,000 – 499,999 | 13 | 18 | 11 | 17 | 14 |
| 500,000 – 999,999 | 13 | 8 | 4 | 6 | 5 |
| 1,000,000+ people | 3 | 14 | 5 | 11 | 9 |
| I don’t know | 5 | 5 | 2 | 2 | 16 |

Supplemental Table 2. Significant relationships between socio-demographics and governmental authorities’ provision of ‘no useful information whatsoever’ on COVID-19

|  | Germany | Norway | Sweden | Switzerland | UK |
| --- | --- | --- | --- | --- | --- |
| Political orientation^2^ | Right-leaning | --- | --- | --- | --- |
| Income^1^ | Lower | --- | --- | --- | --- |
| Education^1,3^ | Lower | --- | --- | --- | --- |
| Sex^1^ | Male | Male | Male | --- | --- |
| Pre-existing conditions^1,4^ | Diabetes | Chronic lung, liver, kidney disease, cancer, smoking |  |  | Kidney disease, cancer, immuno-suppressive conditions |
| Age^2^ | --- | --- | --- | --- | Younger |
| City size^1^ | --- | --- | --- | --- | --- |
| Household size^2^ | --- | --- | --- | --- | --- |
| Born in country^1^ | --- | --- | --- | --- | --- |
| Religious^1,5^ | --- | --- | --- | --- | --- |

**NB:** Significant associations are noted; vacant cells denote non-significant effects.

^1^ Chi-square test on a crosstab

^2^ Independent samples t-test

^3^ Dichotomous variable: bachelor’s degree or higher vs less than a bachelor’s degree

^4^ Self-report; pre-existing conditions included: chronic lung disease, cardiovascular disease, diabetes, liver disease, kidney disease, cancer, immunosuppressive conditions, obesity, and smoking

^5^ ‘Do you consider yourself a member of any religious organisation, spiritual community, or faith group?’

Supplemental Table 3. Significant relationships between socio-demographics and perceived consistency in governmental authorities’ instructions and recommendations on COVID-19

|  | Germany | Norway | Sweden | Switzerland | UK |
| --- | --- | --- | --- | --- | --- |
| Political orientation^1^ | --- | Right-leaning | Left-leaning | Left-leaning | Right-leaning |
| Income^2^ | --- | --- | --- | --- | --- |
| Education^3^ | --- | --- | --- | --- | --- |
| Sex^3^ | Female | --- | --- | --- | --- |
| Pre-existing conditions^3^ | Cardiovascular disease |  | Cardiovascular disease | Chronic lung disease |  |
| Age^1^ | --- | --- | --- | --- | --- |
| City size^2^ | --- | --- | --- | --- | --- |
| Household size^1^ | --- | --- | --- | Larger | Larger |
| Born in country^3^ | --- | --- | --- | --- | --- |
| Religious^3^ | --- | --- | --- | --- | --- |

**NB:** Significant associations are noted; vacant cells denote non-significant effects. The values in the cells are associated with *higher* levels of perceived consistency.

^1^ Bivariate correlations

^2^ Analysis of variance with Tukey post-hoc tests

^3^ Independent samples t-test
