# Supplementary material for: Satisfaction With Governmental Risk Communication Both Increases and Decreases COVID-19 Mitigation Behaviours
Source: Int J Public Health. 2023 Mar 1;68:1604966. doi: 10.3389/ijph.2023.1604966 (PMC10014469; doi:10.3389/ijph.2023.1604966)
Supplement: Supplementary file 3 [file DataSheet1.docx]

Supplemental methods file

Survey design

The sample in each nation was constrained to represent the population of each nation through survey quotas based on: age distribution (by six age categories), sex (female/male), highest level of education completed (three categories of aggregated ISCED levels), and annual income (five to eight categories, based on national census data)^[[1]](#footnote-1)^. The survey was initial constructed in English, then professionally translated into German, Norwegian, Swedish, and French, and second checked by the academic team on our project in Norway, Sweden, Germany, and Switzerland to ensure original meaning was retained. The Swiss survey was made available to respondents in French or German; they could select their preferred language. In addition to the speeding check, we further included an attention filter in which we asked respondents simply to select a specific response option (‘severe risk’) within a list of items on risk perceptions; we eliminated any respondents who did not select the correct answer.

Full question wording of survey items included in our analysis within this study

*Public perceptions of official governmental risk communication:*

1. *Information needs met*: ‘To what extent do you feel that the authorities in your country have met your need for information about risks associated with coronavirus (COVID-19)?   (Note – communication on risks includes advice, guidance, recommendations, and official restrictions, or any other type of formal information about relevant risks.)’
2. *Clear and understandable*: ‘In [country name] coronavirus-related instructions, recommendations and guidance to the public were sometimes changed or modified.   Over time, were the messages that you received on the coronavirus (COVID-19) from official authorities clear and easy to understand?’
3. *Consistent*: ‘Over time, do you feel that the responsible authorities have been consistent in their instructions and recommendations given to the public?’

Questions 2 and 3 above included Likert-style options on a unidirectional scale of 1-5. Question 1 included five options ranging from no useful information whatsoever to overloaded with information.

*Risk perceptions:*

We measured four broad content foci for risk perceptions: personal health risk, public health risks, personal economic risks, and societal risks (economic, political, social). Initial exploratory factor analyses revealed clear pooling of the fifteen measured items into the four categories:

1. *Personal health risk perceptions*: perceived percentage chance in the next three months of (1) getting COVID-19, (2) being hospitalised due to COVID-19, and (3) dying from COVID-19 (Cronbach’s α reliability = 0.88)
2. *Public health risk perceptions*: On a Likert-style scale of 1-5, perceived risk of: (1) more people falling ill in one’s country than elsewhere, (2) more people dying in one’s country than elsewhere, and (3) health services in one’s country becoming overstretched (Cronbach’s α reliability = 0.82)
3. *Personal economic risk perceptions*: perceived percentage chance in the next three months of (1) your financial situation worsening, (2) losing your job, and (3) your relatives losing their jobs (Cronbach’s α reliability = 0.81)
4. *Societal risk perceptions*: On a Likert-style scale of 1-5, perceived risk of COVID-19 leading to, for your country: (1) a deep economic crisis, (2) national debt increase, (3) hardship for small and medium businesses, (4) loss of trust in public authorities, (5) lack of community feeling and solidarity, and (6) children missing school (Cronbach’s α reliability = 0.80)

*Behavioural outcome variables:*

- ‘In [country name], there are variations in how people have protected themselves against the coronavirus. How often do you use the following protection measures?’
  - Keeping the required 'social distance'
  - Getting tested when having symptoms

These items included Likert-style response options on a unidirectional scale of 1-5 (never, rarely, occasionally, most of the time, always).

Note: In additional to the items presented above, we include a separate supplemental file with the full text of the entire survey. The raw data from all 4,206 respondents (SPSS file, .sav) is available from the corresponding author by request.

1. Due to variations in the national census data available for each country, annual *household* income was used as the quota constraint in Germany, Norway, and the UK, whilst annual *personal* income was used in Sweden and Switzerland. We do not use income as a variable to compare across countries in any analysis; we only use it for within country analysis. [↑](#footnote-ref-1)
